# Altered mitochondrial dynamics and function in *APOE4*-expressing astrocytes

**DOI:** 10.1038/s41419-020-02776-4

**Published:** 2020-07-24

**Authors:** Eran Schmukler, Shira Solomon, Shira Simonovitch, Yona Goldshmit, Eya Wolfson, Daniel Morris Michaelson, Ronit Pinkas-Kramarski

**Affiliations:** 1 Department of Neurobiology, Tel-Aviv University, Ramat-Aviv, 69978 Israel; 2 Steyer School of Health Professions, Sackler School of Medicine, Tel-Aviv University, P.O. Box 39040, Tel Aviv, 6997801 Israel; 3 Australian Regenerative Medicine Institute, Monash Biotechnology, 15 Innovation Walk, Clayton, VIC 3800 Australia

**Keywords:** Biochemistry, Neurological disorders

## Abstract

*APOE4* is a major risk factor for sporadic Alzheimer’s disease; however, it is unclear how it exerts its pathological effects. Others and we have previously shown that autophagy is impaired in *APOE4* compared to *APOE3* astrocytes, and demonstrated differences in the expression of mitochondrial dynamics proteins in brains of *APOE3* and *APOE4* transgenic mice. Here, we investigated the effect of *APOE4* expression on several aspects of mitochondrial function and network dynamics, including fusion, fission, and mitophagy, specifically in astrocytes. We found that *APOE3* and *APOE4* astrocytes differ in their mitochondrial dynamics, suggesting that the mitochondria of *APOE4* astrocytes exhibit reduced fission and mitophagy. *APOE4* astrocytes also show impaired mitochondrial function. Importantly, the autophagy inducer rapamycin enhanced mitophagy and improved mitochondrial functioning in *APOE4* astrocytes. Collectively, the results demonstrate that *APOE4* expression is associated with altered mitochondrial dynamics, which might lead to impaired mitochondrial function in astrocytes. This, in turn, may contribute to the pathological effects of *APOE4* in Alzheimer’s disease.

## Introduction

Alzheimer’s disease (AD) is characterized by brain Aβ plaques and neurofibrillary tangles, neuronal loss and cognitive decline. The ε4 allele of *APOE* gene, a major AD risk factor, is associated with increased Aβ deposition and neuronal degeneration^1^. Nonetheless, the mechanisms mediating *APOE4* pathological effects remain unknown^1^. Mitochondrial dysfunction plays a fundamental role in AD pathogenesis^2^ and represents an early event^2^. Mitochondrial dynamics include fusion, fission, and degradation events, linked to mitochondrial function^3,4^. Fusion and fission regulate mitochondrial network size/shape depending on cellular demands. Damaged mitochondria are degraded by selective autophagy, termed mitophagy, following fission^5^. Mitochondrial dynamics were shown to be abnormal in AD brains^6^.

Several proteins are required for mitochondrial fusion, including the mitofusins (Mfn1/Mfn2)^3,4^. This process is triggered by increased mitochondrial membrane potential (MMP) and involves the oligomerization of mitofusins on the outer mitochondrial membrane (OMM)^4^. Mitochondrial fusion is enhanced under stress and high energetic demands, allowing increased bioenergetic efficiency^7^. During mitochondrial fission, cytosolic Drp1 is recruited to mitochondrial receptors (e.g., Fis1)^3,4^, allowing mitochondrial partitioning, turnover and reduction of network size under low energetic demand^4,7^. Mitophagy proceeds mitochondrial fission and is triggered by decreased MMP^5^. It allows the removal of damaged/excessive mitochondria via the lysosome. Several pathways mediate mitophagy, including the PINK1/parkin pathway^5,8^. The mitochondrial kinase PINK1 is stabilized on the OMM leading to the recruitment and activation of parkin (E3 ubiquitin ligase), which ubiquitinates several mitochondrial proteins leading to mitophagy^5,8^. Several studies demonstrated a link between *APOE4* and mitochondrial dysfunction, mainly in neurons^9–11^; yet the effect of *APOE4* on mitochondrial dynamics was less studied. Also, although astrocytes play a fundamental role in AD pathology^12^, little is known about *APOE4* effect on their mitochondria.

Previously, others and we have found that *APOE4* is associated with impaired autophagy in astrocytes^13,14^, which is linked to reduced clearance of Aβ plaques and protein aggregates^13,14^. Also, our recent findings revealed differences in the expression of mitochondrial dynamics proteins in *APOE4* transgenic mouse brains^15^. Here, we further investigated the effect of *APOE4* expression on various mechanistic aspects of mitochondrial dynamics, including mitophagy, and mitochondrial function specifically in astrocytes. Our results show that *APOE4* expression is associated with altered mitochondrial dynamics, namely, reduced mitochondrial fission and parkin-mediated mitophagy, under basal and mitochondrial stress conditions. Furthermore, mitochondrial function is compromised in the *APOE4* astrocytes. Notably, induction of mitophagy by rapamycin restored mitochondrial function. Taken together, the results suggest that impaired mitochondrial dynamics in *APOE4* astrocytes is involved in mitochondrial dysfunction and contribute to AD pathology.

## Materials and methods

The antibodies, buffers, and reagents used are described in the supplementary methods.

### Cell lines and primary astrocytes

The human *APOE3* and *APOE4* targeted-replacement astrocyte cell lines were previously described^16^. The study was conducted according to the NIH Guidelines for Use and Care of Laboratory Animals, following the approval by Animal Care Committee of TAU (#L-04-16-002). Mouse primary astrocytes were prepared and grown as previously described^13^.

### Brain sections preparation and IHC

*APOE3*/*APOE4* targeted-replacement mice were perfused with PBS followed by 4% paraformaldehyde. Brains were removed, post-fixed for overnight at 4 °C in 4% paraformaldehyde followed by 20% sucrose in PBS for 48 h at 4 °C. Coronal serial cryostat sections were cut (20 µm) and stained as described previously^17^. Sections were imaged using fluorescence microscopy (Olympus motorized inverted research microscope Model IX81; ×20 magnifications).

### Lysate preparation, immunoblot and immunoprecipitation

Preparation of cell lysates and Western Blot analysis was performed as previously described^13^. For the immunoprecipitation, agarose beads conjugated to mouse anti-ubiquitin antibodies (Santa Cruz Biotechnology) were used. For no IgG control samples, protein G PLUS-agarose beads (Santa Cruz Biotechnology) were used.

Immunoreactive bands were detected using enhanced chemiluminescence reagent (Immobilon Crescendo substrate, Millipore). Bands were visualized by Amersham Imager 600 within the linear range, employing the Automatic/Semi-autmatic exposure options, which allows optimal exposure times below saturation to enable accurate quantification (according to manufacturer instructions). Target protein band intensities were quantified using the ImageJ software and normalized to loading controls.

### Mitochondrial fractionation

Mitochondrial fractionation of the cells was performed using the ProteoExtract^®^ Cytosol/Mitochondria Fractionation Kit (EMD Millipore), according to manufacturer protocol.

### Analysis of mitochondrial network morphology

*APOE3/APOE4* astrocytes cell line was incubated with 100 nM MitoTracker™ Deep Red FM (MTDR, Invitrogen) for 30 min at 37 °C or infected with Mito-GFP as described below, nuclei were stained with 1 µg/ml Hoechst 33342 and fixed in ice-cold methanol for 15 min (MTDR) or in 4% paraformaldehyde for 30 min at RT (Mito-GFP). Mitochondrial network morphology of individual cells was visualized by Leica TCS SP8 confocal microscope (×63 magnifications) and analyzed using the ImageJ macro tool MiNa^18^.

### Retroviral infection and generation of APOE3/APOE4 cell lines stably expressing Mito-GFP

HEK-293T cells were transfected using the calcium-phosphate method with the following retroviral vectors: pCMV-VSV-G, pCMV-Gag-Pol and pQCXIP containing the EGFP-TA-MAO sequence (Mito-GFP; EGFP-tagged OMM localization signal peptide), which was a gift from Prof. Reuven Stein, Tel Aviv University. Medium containing viral particles was collected and used to infect the *APOE3/APOE4* astrocytes cell line. Astrocytes stably expressing Mito-GFP were selected using 2 µg/ml puromycin.

### Transient transfection with the LC3-EGFP-mRFP vector

Transient transfection of *APOE3/APOE4* astrocytes cell line with the tandem LC3-EGFP-mRFP (Addgene ptfLC3) was performed using Lipofectamine^®^ 2000 reagent (Invitrogen). Nuclei were stained; the cells were fixed and microscopically analyzed as described above.

### Electron microscopy

Preparation of cellular TEM samples from *APOE3/APOE4* astrocytes cell line is described in the supplementary. The samples were analyzed using G-12 Spirit FEI electron microscope.

### MTT, ATP levels, and cell number measurement

MTT and CellTiter-Glo (Promega) assays were used in *APOE3/APOE4* cell line to assess mitochondrial metabolism and ATP levels, respectively, and the results were normalized to cell number using the methylene blue assay according to the manufacturer instructions and as previously described^19,20^.

### Measurement of MMP by flow cytometry

The *APOE3/APOE4* cell line was incubated with either 100 nM MitoTracker™ Red CMXRox (MTR, Invitrogen) or 50 nM MitoTracker™ Deep Red FM (MTDR, Invitrogen) to assess MMP or mitochondrial mass, respectively, by flow cytometry, as described^21–25^. The cells were then trypsinized, washed and resuspended in PBS. MTDR/MTR fluorescence was collected using Stratedigm S1000EXi Flow Cytometer (mCherry and APC filters, respectively), and analyzed using Cyflogic software.

### RNA extraction and qRT-PCR analysis

Total RNA was extracted from *APOE3/APOE4* astrocytes cell line, reverse-transcribed into cDNA and used for RT–PCR as described previously^19^. The primers used are described in the supplementary.

### Medium pH measurement

The medium was collected from *APOE3/APOE4* cell line, placed in sealed tubes, and pH was measured using Sartorius PB-11 Basic Benchtop pH Meter. The number of cells in the cultures was simultaneously measured.

### Statistical analysis

All experiments were performed at least 3 times. Experimental differences were tested for statistical significance using one-sided Student’s *t* test. *p*-value of < 0.05 was considered as significant.

## Results

### Altered mitochondrial dynamics proteins expression and morphology in APOE4 astrocytes

**Fig. 1:**
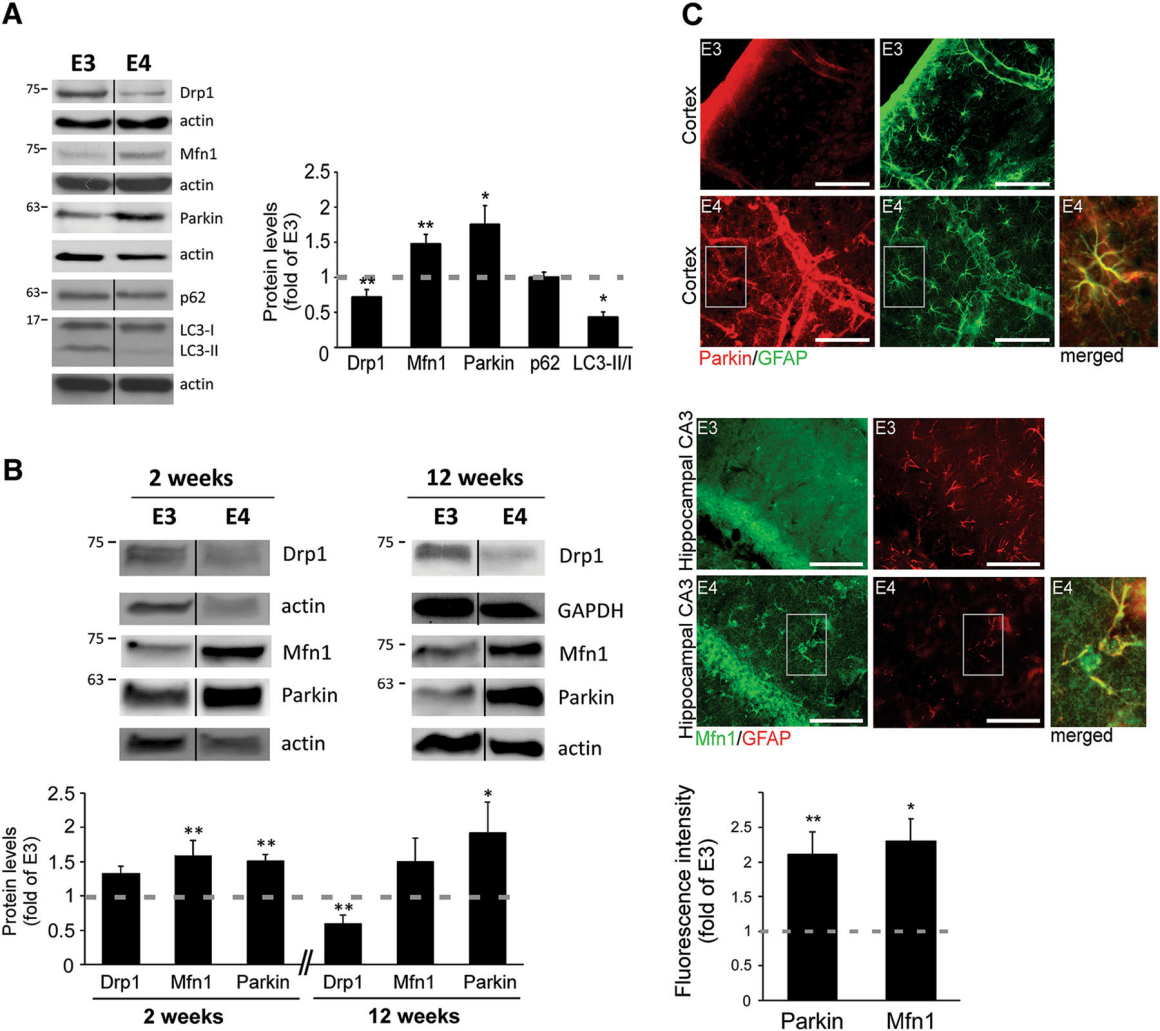
Levels of mitochondrial dynamics proteins in *APOE4* astrocytes. **a** *APOE3/APOE4* astrocytes were subjected to Immunoblot, using the indicated antibodies. *Left panel*, representative results; *right panel*, densitometric analysis of *APOE4* cells as fold of *APOE3* (dashed line; *n* ≥ 3) **b** Primary astrocytes generated from *APOE3/APOE4* targeted-replacement mice were cultured for the indicated time period and subjected to Immunoblot using the indicated antibodies. *Upper panel*, representative results. *Lower panel*, quantification of the results as described in (**a**). **c** Brain sections of *APOE3*/*APOE4* mice (5 months old; *n* = 3 mice/group) were co-stained using anti-GFAP (astrocytic marker) with anti-parkin or anti-Mfn1 antibodies. *Right panel*, co-expression of parkin (red) and GFAP (green) in the cortex area; Mfn1 (green) and GFAP (red) in the hippocampus CA3 area. (scale bars, 50 μm). Magnified inset: merged GFAP with parkin/Mfn1 in *APOE4* astrocytes. *Left panel*, fluorescence intensity was quantified using ImageJ only in GFAP-positive cells, (fold of *APOE3*, dashed line). **a**–**c** Means ± SE; *p < 0.05 and **p < 0.01.

To investigate the link between *APOE4* and mitochondrial dynamics, we first examined the expression of fission, fusion and mitophagy proteins in astrocytes expressing human *APOE4* or *APOE3* (Fig. 1a). As shown, *APOE4* astrocytes exhibit lower Drp1 levels (fission), higher Mfn1 levels (fusion) and increased parkin levels (targets mitochondria for mitophagy, followed by proteasomal/lysosomal degradation^26–29^). In addition, we measured the levels of autophagic markers, SQSTM1/p62 and LC3^30^. *APOE4* had no effect on p62 levels; however, it reduced LC3-II/LC3-I ratio, indicating decreased autophagy^30^. Importantly, similar pattern of these mitochondrial dynamics proteins was detected in primary astrocytes generated from *APOE4* mice (differences in Drp1 levels were detected only in 12 weeks cultures) and brain sections from these mice demonstrated increased levels of parkin and Mfn1, specifically in astrocytes (Fig. 1b, c). These results imply that *APOE4* astrocytes exhibit altered mitochondrial dynamics.

**Fig. 2:**
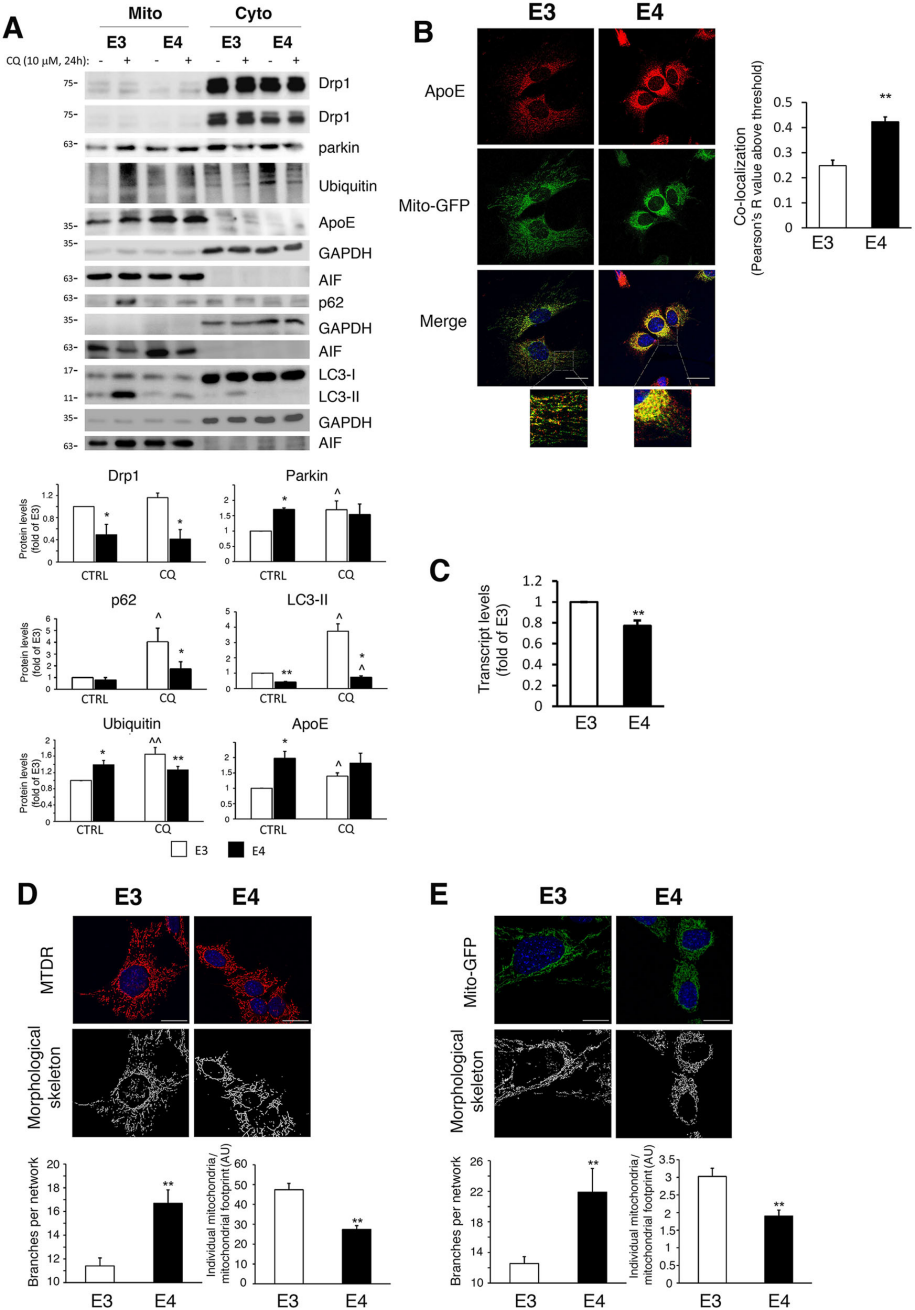
Mitochondrial fraction and mitochondrial morphology in APOE4 astrocytes. **a** *APOE3*/*APOE4* astrocytes were treated with 10 μM chloroquine (CQ) for 24 h. Mitochondrial and cytosolic fractions were subjected to Immunoblot using the indicated antibodies. AIF and GAPDH were used as markers for cytosol and mitochondria, respectively. *Upper panel*, representative results; *lower panel*, densitometric analysis of proteins in the mitochondrial fraction (normalized to AIF; fold of *APOE3*; *n* ≥ 3). **b** *APOE3/APOE4* astrocytes expressing Mito-GFP were stained with anti-apoE antibodies (scale bars 25 μm). *Left panel*, representative results. *Right panel*, co-localization analysis of Mito-GFP (green) and apoE (red) using the Pearson’s *R* value above threshold as calculated by the ImageJ Coloc 2 plugin after thresholding for apoE; *n* ≥ 50 cells; means ± SE; **p < 0.01. **c** *APOE3/APOE4* astrocytes were subjected to real-time PCR for *APOE* expression (fold of *APOE3*; *n* ≥ 3); means ± SE; **p < 0.01. **d**, **e** *APOE3*/*APOE4* astrocytes were incubated with MitoTracker Deep Red (MTDR, **d**) or infected with Mito-GFP (**e**). *Upper panels*, representative images and morphological skeleton analysis as generated by the MiNa tool (Scale bars, 25 μm); *Lower panels*, quantification of branches per network (mean network size) and individual mitochondria relative to the mitochondrial network footprint (*n* ≥ 50 cells). **a**–**e** Means ± SE; *p < 0.05 and **p < 0.01, *APOE3*-expressing com*p*ared with *APOE4*-expressing cells; ^*p* < 0.05, CQ-treated compared to untreated cells.

To further explore mitochondrial dynamics, we examined the mitochondrial fraction of the *APOE3/APO*E4 astrocytes. As shown (Fig. 2a), *APOE3* astrocytes exhibited higher levels of mitochondrial Drp1 and LC3-II, indicating less mitochondrial fission and association of LC3 with mitochondria in *APOE4* astrocytes. Moreover, increased levels of parkin and ubiquitin were detected in *APOE4* astrocytes mitochondria, indicating either increased recruitment of parkin and ubiquitination of mitochondria, or accumulation due to reduced mitochondrial degradation. Therefore, we measured the mitochondrial recruitment of mitophagy/autophagy proteins by examining the mitochondrial fraction following chloroquine treatment^24,30^. As shown in Fig. 2a, increased accumulation (differences between CQ treated and untreated cells) of parkin, p62, LC3-II and ubiquitin was observed in the mitochondrial fraction of the *APOE3* astrocytes following treatment, suggesting their degradation is reduced in *APOE4* astrocytes. Interestingly, although the levels of LC3-II, p62 and ubiquitin in the mitochondrial fraction of CQ-treated *APOE3* cells were higher compared with *APOE4* cells, the levels of parkin did not differ (Fig. 2a), suggesting that the recruitment of parkin to the mitochondria of the *APOE4* astrocytes is intact. Also, fractionation and immunostaining revealed that mitochondrial apoE4 levels are higher compared to apoE3 (Fig. 2a, b), which is not due to increased *APOE4* mRNA synthesis (Fig. 2c). Chloroquine induced accumulation only of mitochondrial apoE3 (Fig. 2a), suggesting increased accumulation of mitochondrial apoE4.

To further explore whether *APOE4* is associated with decreased mitochondrial fission and degradation, the mitochondrial network morphology was visualized using MitoTracker Deep Red staining or Mito-GFP expression. As demonstrated (Fig. 2d, e), the mitochondrial network of the *APOE4* astrocytes is indeed more hyperfused (exhibits more branching and less individual mitochondria).

### APOE4-expressing astrocytes exhibit impaired mitophagy

**Fig. 3:**
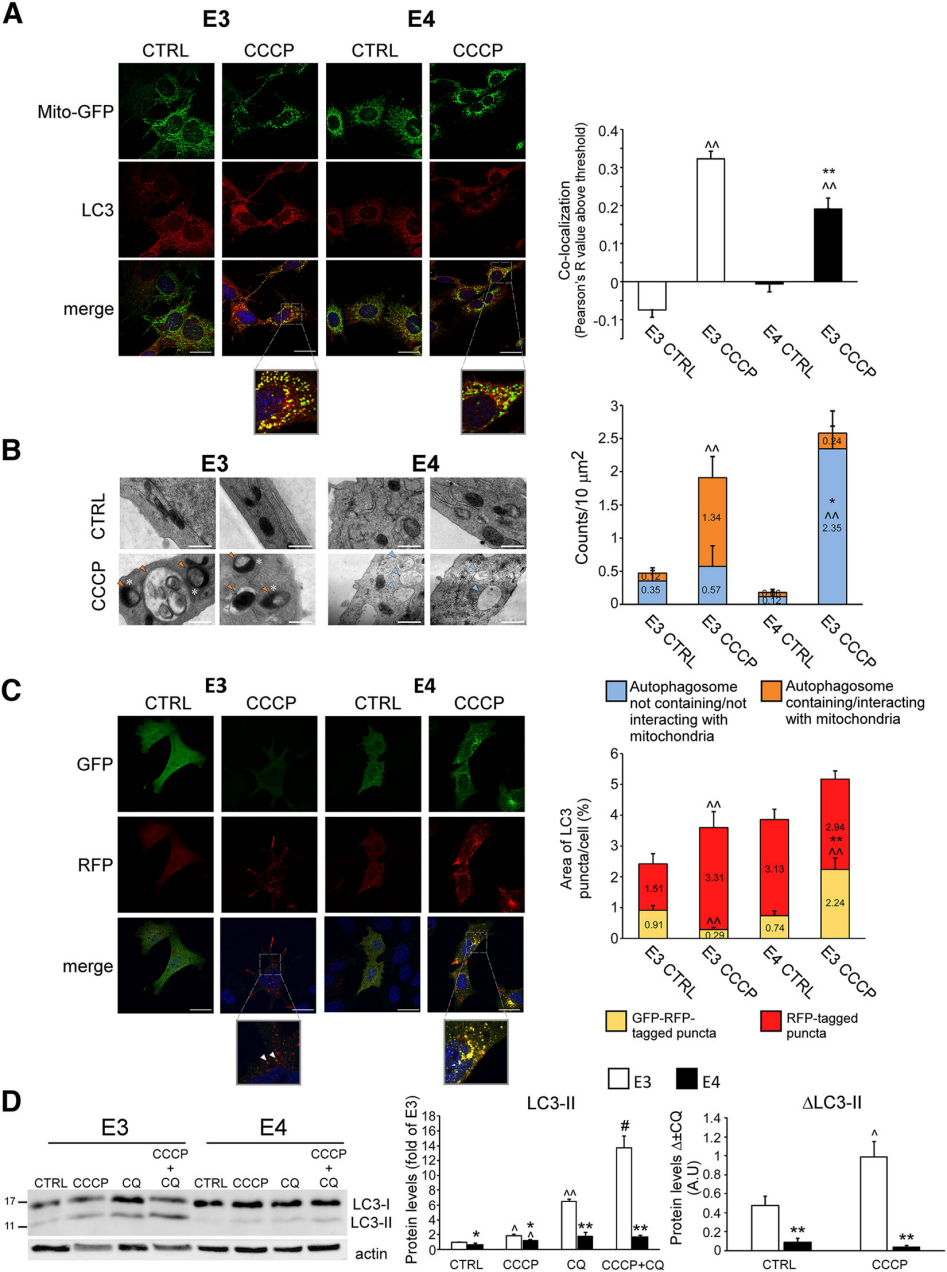
CCCP-induced mitophagy in *APOE3/APOE4* astrocytes. **a** *APOE3/APOE4* astrocytes expressing Mito-GFP were treated with 15 μM CCCP for 1 h and stained with anti-LC3 antibodies (scale bars 25 μm). *Left panel*, representative results. *Right panel*, co-localization analysis of Mito-GFP (green) and LC3 puncta (red) using the Pearson’s R value above threshold as calculated by the ImageJ Coloc 2 plugin after thresholding for LC3 puncta; *n* ≥ 50 cells/treatment; means ± SE; **p < 0.01, *APOE3* compared with *APOE4*; ^^*p* < 0.01, CCCP-treated compared to untreated cells. **b** *APOE3/APOE4* astrocytes were treated with 25 μM CCCP for 4 h and analyzed by electron microscopy. *Left panel*, representative micrographs; *right panel*, the number of autophagosomes containing/interacting (orange arrows) or not (blue arrows) with mitochondria was measured per 10 μm^2^ fields (scale bars, 500 nm); *n* ≥ 10 fields/treatment; means ± SE; *p < 0.05 and **p < 0.01, *APOE3* compared with *APOE4*; ^^*p* < 0.01, CCCP-treated compared to untreated cells.; white asterisks, spheroid/ring-shaped mitochondria (presumably mitochondria interacting with autophagosome/lysosome) **c** *APOE3/APOE4* astrocytes expressing LC3-EGFP-mRFP were treated with 10 μM CCCP for 4 h. *Left panel*, representative results (scale bars 25 μm)*; right panel*, ImageJ analysis of %area of LC3-EGFP-mRFP (arrows) or LC3-mRFP (arrow heads) positive puncta; *n* ≥ 50 cells/treatment; means ± SE; *p < 0.05 and **p < 0.01, *APOE3*- compared with *APOE4*; ^^*p* < 0.01, CCCP-treated compared to untreated cells. **d** *APOE3/APOE4* astrocytes were treated with 10 μM chloroquine (CQ) for 48 h, with or without 7.5 μM CCCP for the last 24 h. Levels of LC3 were determined by Immunoblot. *Left panel*, representative results; *right panel*, densitometric analysis of the results is presented as fold of *APOE3* (left graph) and as the difference between measured values of LC3 levels with or without CQ (right graph, Δ ± CQ); *n* = 3; means ± SE; *p < 0.05 and **, *APOE3* compared with *APOE4*; ^*p* < 0.05 and ^^*p* < 0.01, treated com*p*ared with untreated cells, ##*p* < 0.01, combined CCCP and CQ treatment compared to each treatment alone.

The reduced fission/mitophagy observed in the *APOE4* astrocytes may result from abnormal metabolic state, or may represent mitophagy impairment. Thus, we next examined the effect of mitochondrial damage induced by mitochondrial uncoupler CCCP^8^ on mitochondrial dynamics. CCCP markedly increased LC3 puncta (red), as well as their co-localization with mitochondria (green), suggestive of mitophagy induction (Fig. 3a), which was higher in *APOE3* astrocytes. Likewise, EM analysis revealed that in the *APOE3* astrocytes, CCCP increased the number of autophagosomes containing mitochondria and the formation of spheroid mitochondria, which represents mitochondria interacting with autophagosomes/lysosomes^31,32^ (Fig. 3b). In contrast, in *APOE4* astrocytes the treatment induced significant formation of autophagosomes that do not contain mitochondria (Fig. 3b). Furthermore, using the tandem LC3-GFP-RFP reporter^30^, we found that following CCCP treatment, ~90% of autophagosomes in the *APOE3* astrocytes were fused with lysosomes, compared to ~55% in the *APOE4* astrocytes (Fig. 3c). Accordingly, *APOE3* astrocytes treated with CCCP combined with chloroquine showed increased accumulation of LC3-II compared to *APOE4* astrocytes, indicating higher autophagic flux^30^ (Fig. 3d). Thus, damaged mitochondria recognition by autophagosomes and their delivery to lysosomes is hampered in *APOE4* astrocytes. Consistently, in *APOE3/APOE4* astrocytes stably expressing Mito-GFP, treatment with CCCP resulted in decreased number of branches per network, which was more profound in the *APOE3* astrocytes (Fig. S1a).

We next studied the effect of CCCP on fission/mitophagy at the protein level. As shown, CCCP treatment induced a dose-dependent decrease of parkin and Mfn1 levels (indicating their degradation) and increased LC3-II/LC3-I ratio (autophagosomes formation), consistent with mitophagy induction (Fig. S1b); yet, the effect was more pronounced in the *APOE3* cells. In addition, 12 µM CCCP treatment reduced p62 levels only in the *APOE3* astrocytes, indicating increased p62 degradation in these cells (Fig. S1b). Examination of the mitochondrial fractions revealed that CCCP treatment increased mitochondrial Drp1 levels only in the *APOE3* astrocytes, and elevated mitochondrial LC3-II, which was more prominent in the *APOE3* astrocytes (Fig. S1c). This suggests that following treatment, less fission and association of LC3-II with damaged mitochondria occurs in *APOE4* astrocytes. Taken together, the results further demonstrate dysfunctional fission and mitophagy in the *APOE4* astrocytes following mitochondrial damage.

### APOE4-expressing astrocytes exhibit altered synthesis, ubiquitination and proteasomal/lysosomal degradation of mitochondrial dynamics proteins

**Fig. 4:**
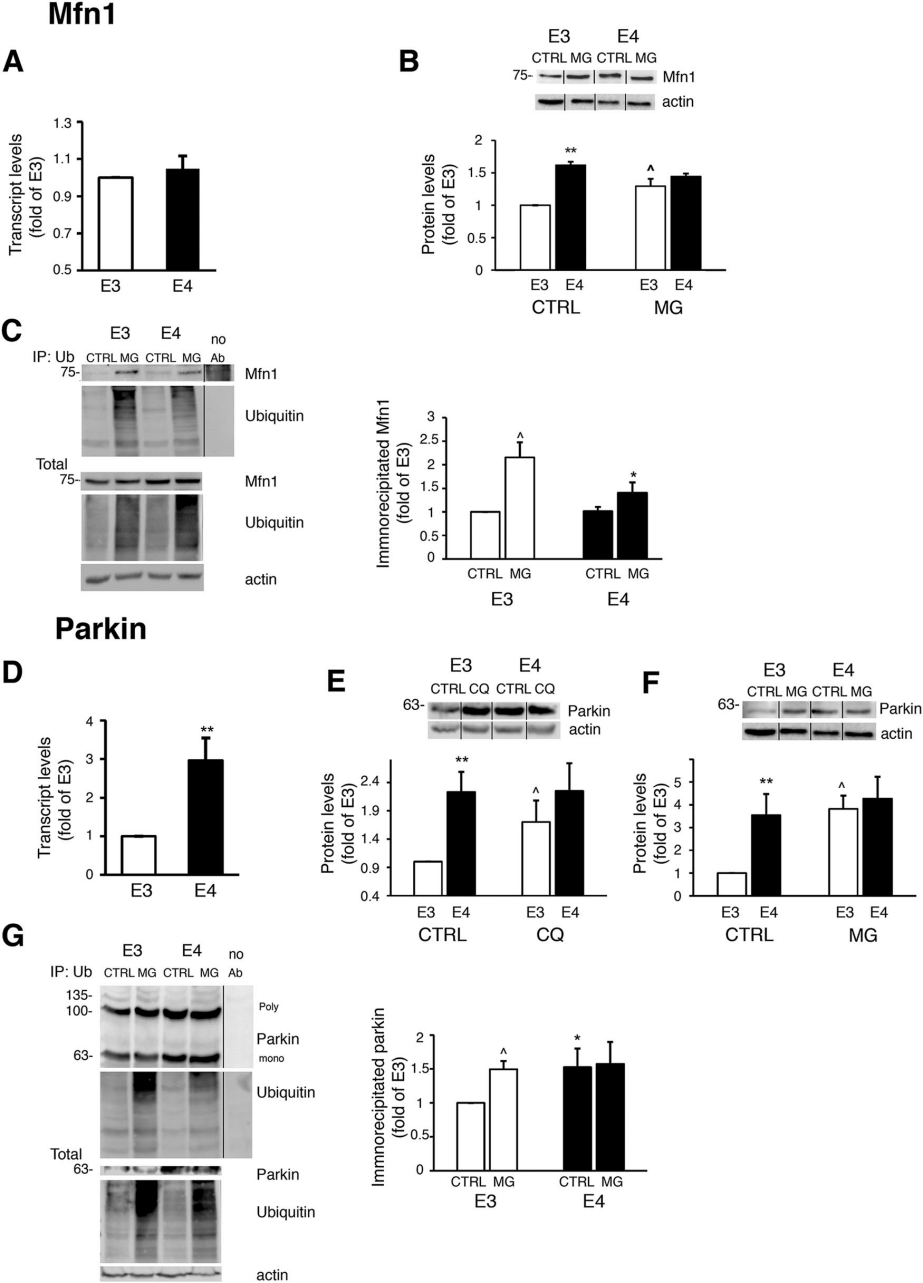
Synthesis/degradation of Mfn1 (a-c) and Parkin (d-g) in *APOE3/APOE4* astrocytes. **a**, **d** *APOE3*/*APOE4* astrocytes were subjected to real-time PCR of *MFN1* (**a**) or *PARK2/PRKN* (parkin) (**d**) expression (fold of *APOE3*; *n* ≥ 3). **b** Cells were treated with 1 μM MG-132 (MG) for the indicated times and the levels of Mfn1 were determined by Immunoblot. *Upper panel*, representative results*; lower panel*, densitometric analysis of Mfn1 levels is presented as fold of *APOE3* (left graph) (*n* ≥ 3). **c**, **g** Cells were treated with 1 μM MG-132 for 8 h and cell lysates were subjected to immunoprecipitation with anti-ubiquitin antibodies. *Left panel*, representative results. *Right panel*, densitometric analysis of immunoprecipitated ubiquitinated Mfn1 (**c**) or ubiquitinated parkin (**g**) (fold of *APOE3*; *n* ≥ 3). **e**, **f** Cells were treated with 10 μM chloroquine (CQ; **e**) or 1 μM MG-132 (MG; **f**) for 24 h or 8 h, respectively, and the parkin levels were determined by Immunoblot. *Upper panel*, representative results*; lower panel*, densitometric analysis of parkin levels (fold of *APOE3*, *n* ≥ 3). **a**–**g** Means ± SE; *p < 0.05 and **p < 0.01, *APOE3* compared to *APOE4*; ^*p* < 0.05 and ^^*p* < 0.01, CQ/MG-132 treated compared to untreated cells.

We next characterized the levels of synthesis, ubiquitination and turnover of mitochondrial dynamics proteins. According to qRT-PCR (Fig. 4a), the observed elevation of Mfn1 protein in *APOE4* astrocytes (Fig. 1) is not due to increased *MFN1* transcription. However, blockage of proteasomal degradation (MG-132), led to accumulation of Mfn1, only in the *APOE3* astrocytes (Fig. 4b). Therefore, we next measured the levels of Mfn1 ubiquitination (Fig. 4c). Following MG-132 treatment the total amount and accumulation of ubiquitinated Mfn1 was higher in *APOE3* compared to *APOE4* astrocytes. During mitophagy, Mfn1 undergoes ubiquitination by parkin followed by proteasomal degradation, which suppresses mitochondrial fusion and allows the removal of mitochondria from the network^8^. Thus, it appears that the basal differences of Mfn1 levels observed in *APOE4* result from reduced Mfn1 ubiquitination and proteasomal degradation, possibly contributing to their hyperfused mitochondrial network (Fig. 2d, e).

The reduced levels of Drp1 in *APOE4* astrocytes (Fig. 1) could result from decreased *DNM1L* (Drp1) mRNA synthesis (Fig. S2a), while degradation does not appear to contribute to this difference, since MG-132/chloroquine had no effect of Drp1 protein levels (Fig. S2b, c).

Interestingly, according to qRT-PCR, the increased parkin levels in *APOE4* astrocytes are at least partially due to increased *PARK2*/*PARKN* mRNA synthesis (Fig. 4d). Additionally, treatment with chloroquine/MG-132 led to parkin accumulation only in the *APOE3* astrocytes (Fig. 4e, f), indicating its reduced mitophagic and proteasomal degradation, respectively, in the *APOE4* astrocytes. Proteasomal degradation of parkin was demonstrated during mitophagy due to autoubiquitination, halting its activity^28,29,33,34^. Immunoprecipitation assay revealed that the levels of ubiquitinated parkin (Fig. 4g) are higher in *APOE4* astrocytes, and that treatment with MG-132 induces accumulation of ubiquitinated parkin only in *APOE3* astrocytes (Fig. 4g). Therefore, the results indicate that parkin undergoes ubiquitination in *APOE4* astrocytes, yet it is not degraded by the proteasome. It is possible that the high levels of ubiquitinated parkin in the *APOE4* astrocytes interfere with its ability to ubiquitinate other proteins (e.g., Mfn1), resulting in their reduced labeling for proteasomal/mitophagic degradation. Indeed, the levels of two other mitochondrial parkin substrates, namely Tom40 and Tom20^35^, exhibited higher levels in *APOE4* astrocytes (Fig. S2d). The elevated levels of Tom40 were not due to mRNA synthesis (Fig. S2e), and following MG-132 treatment, Tom40 accumulated only in *APOE3* astrocytes (Fig. S2f), suggesting that reduced ubiquitination of Tom40 underlies its increased levels in *APOE4* astrocytes. Collectively, the results indicate that parkin activity is altered in the *APOE4* astrocytes.

### APOE4 astrocytes exhibit impaired mitochondrial function

**Fig. 5:**
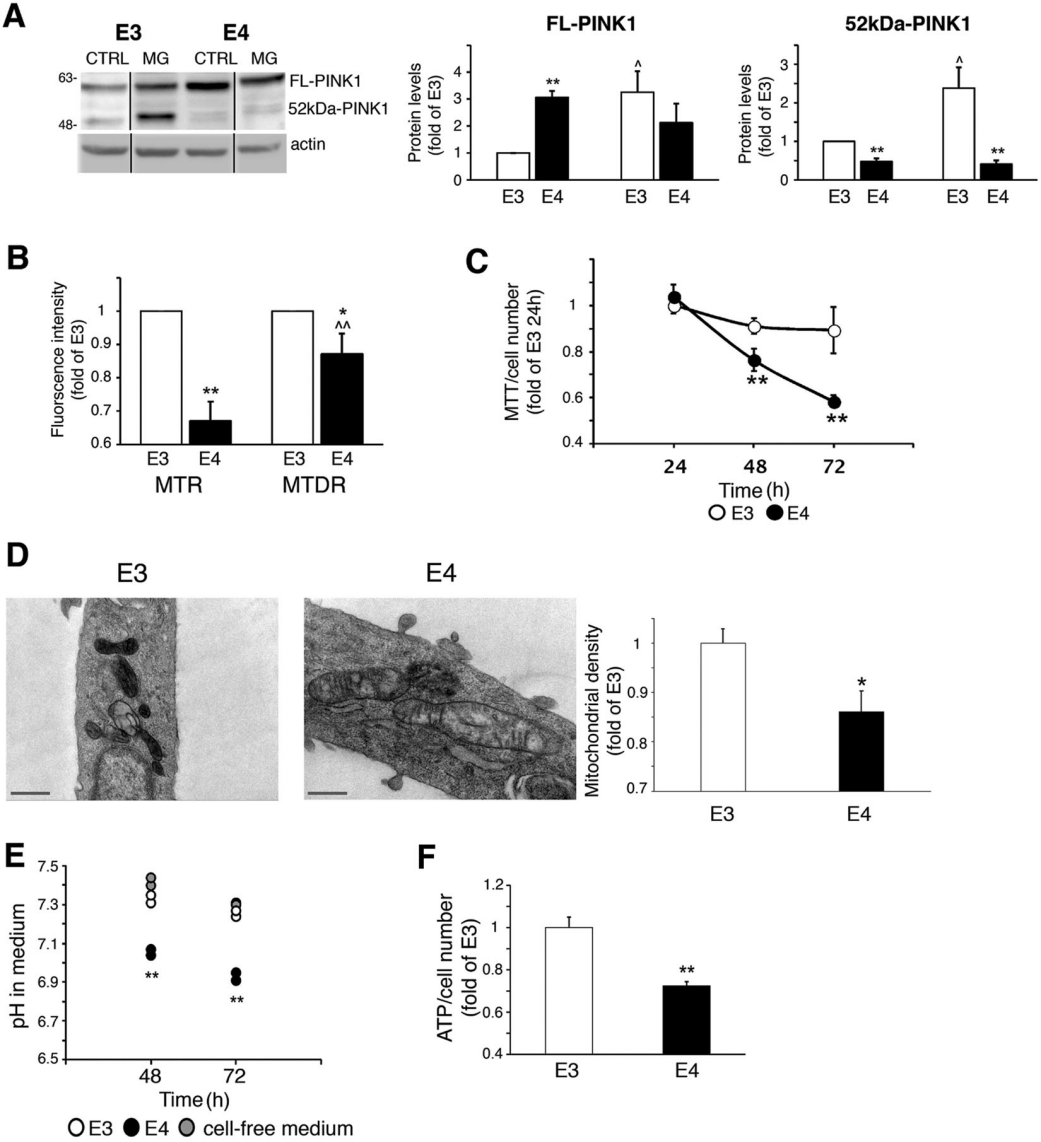
Mitochondrial function in *APOE3/APOE4*. **a** Cells were treated with 1 μM MG-132 (MG) for 8 h and PINK1 levels were determined by Immunoblot. *Left panel*, representative results*; Right panel*s, densitometric analysis of PINK1 levels (fold of *APOE3*; *n* ≥ 3). FL-PINK1, full-length PINK1; 52kDa-PINK1, cleaved PINK1; *p < 0.05 and **p < 0.01, *APOE3* compared to *APOE4*; ^*p* < 0.05 and ^^*p* < 0.01, treated compared to untreated cells. **b** Cells were stained with MitoTracker Red (MTR) or MitoTracker Deep Red (MTDR), and fluorescence was measured (fold of *APOE3*; *n* ≥ 3); *p < 0.05 and **p < 0.01, *APOE3*-expressing compared to *APOE4* cells; ^*p* < 0.05, MTDR compared with MTR. **c**, **g** Cells were subjected to MTT assay normalized to cell number at the indicated time points (**c**) (*n* = 6/treatment). **d** Cells were processed and analyzed by electron microscopy. *Left panel*s, representative results; *right panel*, analysis of mitochondrial density was measured using ImageJ (fold of *APOE3*; *n* ≥ 20 mitochondria; scale bars, 500 nm); means ± SE; **p < 0.01. **e**, **f** Cells were cultured for 48 h, after which, media pH (**e**) or ATP levels (**f**) were measured (*n* ≥ 3); means ± SE; **p < 0.01, *APOE3*-expressing compared to *APOE4*-expressing cells.

Since mitochondrial dynamics are related to mitochondrial quality control, we next investigated several aspects of mitochondrial function. We first focused on PINK1, which accumulates on the OMM following MMP reduction, while it undergoes cleavage in healthy mitochondria due to proteasomal degradation^5,8^. As shown, *APOE4* astrocytes express higher levels of full-length PINK1 (Fig. 5a), which does not result from increased mRNA synthesis (Fig. S3a), and lower levels of cleaved PINK1 compared to *APOE3* astrocytes, which were increased following MG-132 treatment only in *APOE3* astrocytes (Fig. 5a). Collectively, the results indicate that PINK1 cleavage is reduced in *APOE4* astrocytes, indicating reduced MMP. Likewise, the fluorescence of the MMP-sensitive MitoTracker Red^22,23^ was significantly lower in the *APOE4* astrocytes (Fig. 5b). This probably does not reflect reduced mitochondrial content in the *APOE4* astrocytes, since their fluorescence of MitoTracker Deep Red, which is less sensitive to MMP^21,24,25^, was significantly greater compared to MitoTracker Red (Fig. 5b); and the levels of several abundant mitochondrial proteins and transcripts were similar in the *APOE3/APOE4* astrocytes (Fig. S3b, c).

Similarly, the *APOE4* astrocytes exhibit reduced mitochondrial metabolism as measured by MTT^20,36^ (Fig. 5c), and by less dense cristae, which is another indicator of impaired mitochondrial activity^37,38^ (Fig. 5d). Interestingly, the *APOE4* astrocytes also exhibit lower medium pH, suggestive of increased compensatory glycolysis^39^ (Fig. 5e) and reduced ATP levels (Fig. 5f).

### Rapamycin induces mitophagy and restores mitochondrial function in APOE4-expressing astrocytes

We next examined whether non-toxic mitophagy induction could counteract mitochondrial dysfunction in *APOE4* astrocytes. For this aim we have used rapamycin, which promotes autophagosome formation^30^, and was shown to induce mitophagy by upregulating the parkin/PINK1 pathway^25,40–42^.

**Fig. 6:**
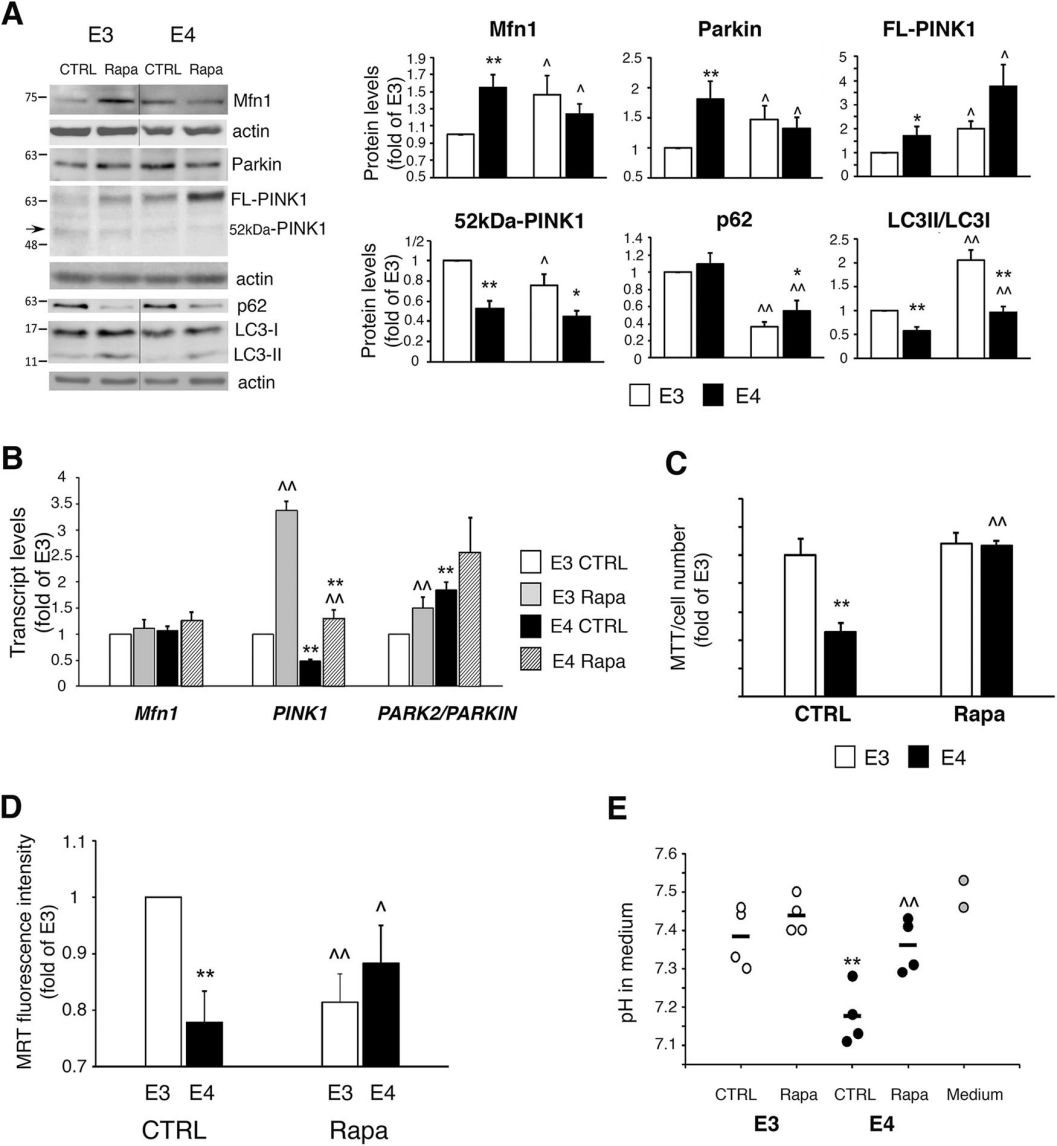
Effect of rapamycin on *APOE3/APOE4* astrocytes. **a** *APOE3/APOE4* astrocytes were treated with 150 nM rapamycin (Rapa) for 48 h and subjected to Immunoblot. *Left panels*, representative results. *Right panels*, densitometric analysis of protein levels (fold of *APOE3*; *n* ≥ 3). **b** Cells were treated with 150 nM rapamycin for 24 h and subjected to real-time PCR (fold of *APOE3*; *n* ≥ 3). **c** Cells were treated with 150 nM rapamycin for the last 24 h (72 h culture) and subjected to MTT assay normalized to cell number (fold of *APOE3*; *n* = 6/treatment). **d** Cells were treated with 150 nM rapamycin for 72 h, followed by staining with MitoTracker Red (MTR). Fluorescence intensity of MTR was measured using flow cytometry (fold of *APOE3*; *n* ≥ 3/treatment). **e** Cells were treated with 150 nM rapamycin for 72 h, after which, media pH was measured (*n* = 3/treatment). **a**–**e** Means ± SE; *p < 0.05 and **p < 0.01, *APOE3* compared with *APOE4*; ^*p* < 0.05 and ^^*p* < 0.01, treated compared with untreated cells.

Rapamycin treatment increased LC3-II/LC3-I ratio and decreased p62 levels, in both the *APOE3/APOE4* astrocytes (Fig. 6a), indicating enhanced autophagosome formation and cargo degradation. Furthermore, in the *APOE4* astrocytes, rapamycin decreased Mfn1 and parkin levels, which is not the result of reduced gene expression (Fig. 6a, b), and is indicative for mitophagy induction (Fig. S1b). Accordingly, rapamycin decreased *APOE4* astrocytes’ mitochondrial content, as demonstrated by MitoTracker Deep Red (Fig. S3d). Rapamycin had no effect on cleaved PINK1 levels in *APOE4* astrocytes, indicating no reduction in MMP, yet it elevated FL-PINK1 levels (Fig. 6a), which probably results from enhanced *PINK1* mRNA synthesis (Fig. 6b). Notably, in the *APOE4* astrocytes, rapamycin restored mitochondrial metabolism (MTT), augmented MMP (MitoTracker Red), and corrected the altered medium pH without affecting cell number (Fig. 6c–e). Taken together, these results demonstrate that rapamycin restores mitochondrial function in the *APOE4* astrocytes.

## Discussion

Changes in mitochondrial dynamics and mitochondrial dysfunction are recognized as hallmarks of AD, and although mitochondrial deterioration in astrocytes occurs early in the disease development, the mechanism is vague^12^. Among the risk factors for AD is the *APOE4* allele^1^; however, its effects on mitochondrial dynamics and dysfunction are unclear. Previously, it was shown that *APOE4* is associated with impaired autophagy in astrocytes^13,14^, as well as with changes in mitochondrial dynamics proteins expression in transgenic mice brains^15^. This prompted us to further explore the mechanisms involved in the effect of *APOE4* on mitochondrial dynamics and function specifically in astrocytes.

Using primary astrocytes and GFAP-stained brain sections generated from *APOE3/APOE4* targeted-replacement mice, we demonstrated differences in mitochondrial dynamics proteins expression similar to those observed in *APOE3* and *APOE4*-expressing immortalized astrocytes. We further demonstrated that *APOE4* expression in astrocytes is associated with altered synthesis, recruitment, ubiquitination and degradation of mitochondrial dynamics proteins. Drp1 protein and mRNA levels and its mitochondrial recruitment, are decreased in *APOE4* astrocytes, indicating reduced mitochondrial fission. Mitochondrial fusion is mediated by mitofusins, which are regulated by transcription and protein modification, including ubiquitination^3^. During mitophagy, parkin ubiqitinates Mfn1, leading to its proteasomal degradation and fusion inhibition^8,43^. We found that Mfn1 ubiquitination/degradation is reduced in *APOE4* astrocytes, leading to increased Mfn1 levels and fusion, possibly interfering with mitophagy.

Parkin labels mitochondria for mitophagy by ubiquitinating specific mitochondrial proteins; parkin is then degraded by the proteasome/lysosome due to autoubiquitination^26–29^. *APOE4* astrocytes exhibit increased levels of parkin, possibly due to reduced degradation of ubiquitinated mitochondrial parkin. This inefficient turnover of parkin might impair its activity, leading to deficient labeling of mitochondria for mitophagy. In fact, abnormal parkin ubiquitination was shown to prevent its proteasomal degradation and impede parkin-mediated mitophagy^44^. Consistently, the ubiquitination/proteasomal degradation of Mfn1 and Tom40^8,35^, as well as of total mitochondrial ubiquitin, are reduced in the *APOE4* astrocytes, further indicating impaired parkin activity. Moreover, the interaction of the autophagic proteins LC3-II and p62 with the mitochondria, and the lysosomal degradation of mitochondrial LC3-II and p62 are reduced in *APOE4* astrocytes. Therefore, the results indicate that mitochondrial dynamics are altered in *APOE4* astrocytes, and specifically suggest reduced parkin-mediated mitophagy.

To investigate whether these findings represent mitophagy impairment, we examined mitochondrial dynamics following induction of mitochondrial damage (CCCP), which normally activates mitophagy^8^. Mitophagy induction following damage was impaired in *APOE4* compared to *APOE3* astrocytes. Consistently, other studies have demonstrated impaired mitophagy and parkin accumulation in AD neurons^45,46^. Interestingly, we also detected less autophagosomes-lysosomes fusion in *APOE4* astrocytes following CCCP treatment, which is consistent with other lysosomal dysfunctions associated with *APOE4*^47^. The mechanism by which *APOE4* affects mitochondrial dynamics is yet to be investigated; it may involve a detrimental interaction of apoE4 itself with the mitochondria, as was shown in neurons^10,11^ and is consistent with our findings. Alternatively, apoE4 may affect mitochondrial dynamics indirectly, by interfering with the autophagy/lysosomal machineries, which affects upstream processes (e.g., mitochondrial fission and labeling) as was demonstrated in other neurodegenerative disorders^47,48^.

The hyperfused mitochondrial network morphology observed in *APOE4* astrocytes may reflect reduced mitophagy and fission, and correlates with findings from AD fibroblasts^6^. Yet, we did not detect a greater mitochondrial mass in *APOE4* astrocytes, suggesting that their mitochondrial network is in a static hyperfused state^49^; i.e., less fission/degradation occurs together with reduced mitochondrial biogenesis. Therefore, less quality control and mitochondrial turnover is possible, leading to increased population of dysfunctional mitochondria^50^. Accordingly, we found impaired mitochondrial activity in *APOE4* astrocytes. Possibly, this mitochondrial dysfunction reduces energy production, which, in turn, represses Drp1 expression and mitochondrial fission to maintain high mitochondrial volume^51,52^.

Assuming that impaired removal of damaged mitochondria underlies *APOE4* astrocytes mitochondrial dysfunction, it is plausible that mitophagy induction, without causing mitochondrial damage, will improve mitochondrial function. Indeed, rapamycin treatment, which was shown to stimulate mitophagy^40,41,53^, partially improved *APOE4* astrocytes’ mitochondrial function. Hence, non-toxic mitophagy might induce efficient removal of dysfunctional mitochondria, therefore improving mitochondrial activity, which in turn, improves AD-related function in *APOE4* astrocytes.

Taken together, our results show that *APOE4* astrocytes exhibit altered mitochondrial dynamics, specifically impaired mitophagy. In addition, these cells display a mitochondrial dysfunction that might be linked to the impaired mitophagy. Thus, the results suggest that altered mitochondrial dynamics in astrocytes plays a role in the pathological effects of *APOE4*.

## Supplementary information

Figure S1

Figure S2

Figure S3

Supplementary Materials and Methods
