# Supplementary material for: Altered mitochondrial dynamics and function in APOE4-expressing astrocytes
Source: Cell Death Dis. 2020 Jul 24;11(7):578. doi: 10.1038/s41419-020-02776-4 (PMC7382473; doi:10.1038/s41419-020-02776-4)

**Supplementary Materials and Methods**

**Materials and buffers**

The following antibodies were used: rabbit anti-Drp1, mouse anti-parkin, rabbit anti-GAPDH and rabbit anti-LC3A/B were from Cell Signaling Technology; rabbit anti-Mfn1, rabbit anti-Tom40, mouse anti-Tom20, mouse anti-Tim23, mouse anti-Drp1, mouse anti cytochrome c, mouse anti-apoE, mouse anti-ubiquitin, mouse anti-COX5A and goat anti-AIF were from Santa Cruz Biotechnology; rabbit anti-p62/SQSTM1 and mouse anti-parkin was from MBL International; rabbit anti-PINK1 was from Novus Biologicals; rabbit anti-LC3B and mouse anti-tubulin were from Sigma-Aldrich; mouse anti-actin was from MP Biomedicals; mouse anti-GFAP was from EMD Millipore.

The following reagents were from Sigma-Aldrich: carbonyl cyanide m-chlorophenyl hydrazine (CCCP), chloroquine, MG-132; and rapamycin was from Cayman Chemical.

Lysates were orepared using solubilization buffer that contained 50mM HEPES (pH 7.5), 150mM NaCl, 1% Triton X-100, 1mM EGTA, 1mM EDTA, 1.5mM MgCl2, 10% glycerol, 0.2mM sodium vanadate, inhibitors cocktail (EMD Millipore, 539131). TBST buffer contained 0.02 M Tris-HCl (pH 7.5), 0.15 M NaCl, and 0.05% Tween 20. HNTG buffer contained 20 mM HEPES (pH 7.5), 150 mM NaCl, 0.1% Triton X-100, and 10% glycerol.

# Primers used for qRT-PCR analysis

The primers used to detect (murine) mRNA are as follows: *MFN1* (Mfn1) forward, 5'-GCTGTCAGAGCCCATCTTTC-3' and reverse, 5'-CAGCCCACTGTTTTCCAAAT-3'; *DNML1* (Drp1) forward, 5'-AAACTCCTATCACGCTCATCA-3' and reverse, 5'-CTCATCCTCCACGCATCCT-3’; *PARK2* (Parkin) forward, 5’-GGAGGAGGCCTGGATGACT-3’ and reverse, 5’-GACAAACACTATCATGGTCACCG-3’; *PINK1* (PINK1) forward, 5’-CTTATAGGAAAGGGCCCGGATGTCG-3’ and reverse, 5’- GATGATGTTAGGGTGTGGGGCAAGC-3’; *mtND1* (Mitochondrially Encoded NADH: Ubiquinone Oxidoreductase Core Subunit 1) forward, 5’-CGCCCTAACAACTATTATCTTCC-3’ and reverse, 5’-GAAGCGTGGATAAGATGCTC-3’; *mtATP6* (Mitochondrially Encoded ATP Synthase Membrane Subunit 6) forward, 5’-AAATATTAGCCCACCAACAG-3’ and reverse, 5’-CTAGGAGGGTGAATACGTAG-3’; *GAPDH* forward, 5'-AAGGTCATCCCAGAGCTGAA-3' and reverse, 5'-GCCATGAGGTCCACCACCCT-3'. GAPDH was used as a reference gene for normalization of relative mRNA expression.

# Preparation of TEM samples

For the preparation of TEM samples, *APOE3/APOE4* astrocytes cell line was fixed for 2 h in Karnovsky fixative and washed with 0.1 M sodium cacodylate buffer. The cells were post-fixed in 1% OsO_4_, 0.5% K_2_Cr_2_O_7_, 0.5% K_4_[Fe(CN)_6_] in 0.1 M cacodylate-buffer (pH 7.4) for 1 h at room temperature, then washed twice with 0.1 M cacodylate-buffer, followed by rinsing with DDW three times. Cells were then stained with 2% uranyl-acetate for 1 h, washed with DDW, dehydrated in ethanol and embedded in Epon EMbed 812 (EMS). The resin was polymerized at 60 °C for 24 h. Ultra-thin sections (90-70 nm) were obtained with a Leica Ultracut Ultramicrotome. The sections from all groups were stained with Pb_3_C_12_H_10_O_14_ simultaneously.

**Legends to supplementary figures**

**Figure S1: Mitochondrial morphology and the levels of mitochondrial dynamics proteins in *APOE3-* and *APOE4-*expressing astrocytes following CCCP treatment.** **A.** *APOE3/APOE4* astrocytes stably expressing Mito-GFP were treated with 15 μM CCCP for 1 h. *Left panels*, representative images and morphological skeleton analysis as generated by the MiNa tool (Scale bars, 25 μm); *right panels*, quantification of branches per network (upper graph; mean network size) and percentage of branches consumed following treatment (lower graph) (n=40 cells) **B.** *APOE3*- and *APOE4*-expressing astrocytes were treated with CCCP for 24 h at the indicated concentrations. The levels of the indicated proteins were determined by Immunoblot. *Left panel,* representative results*; right panel*, densitometric analysis (fold of *APOE3*-expressing cells; n > 3) **B.** *APOE3*- and *APOE4* astrocytes were treated with 25 μM CCCP for 4 h, followed by cell fractionation. Mitochondrial and cytosolic fractions were subjected to Immunoblot using the indicated antibodies. The levels of AIF and GAPDH were used as markers for cytosol and mitochondria, respectively. *Left panel*, representative results; *right panel*, densitometric analysis of proteins in the mitochondrial fraction (normalized to AIF, fold of *APOE3-*expressing cells; n > 3) **(A-C)** means SE; * *p* < 0.05 and ** *p* < 0.01, *APOE3* compared with *APOE4* cells; ^ *p* < 0.05 and ^^ *p* < 0.01, CCCP-treated compared to untreated cells.


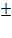


**Figure S2: Levels of DRP1 and Tom proteins in *APOE3/APOE4* astrocytes A..** *APOE3* and *APOE4* astrocytes were subjected to real-time PCR of *DNM1L* (Drp1; fold of *APOE3*-expressing cells; n > 3). **B-C.** Cells were treated with 1 μM MG-132 (MG) (B) or 10 μM chloroquine (CQ)(C) for 8 h or 24 h, respectively, and the levels of Drp1 were determined by Western Blot analysis. *Upper panel*s, representative results*; lower panels*, densitometric analysis of Drp1 levels (fold of *APOE3*-expressing cells; n > 3). D. *APOE3-* and *APOE4*-expressing astrocytes were lysed and subjected to Immunoblot of Tom40 and Tom20. *Left panel,* representative results*; right panel*, densitometric analysis (fold of *APOE3*-expressing cells; n > 3) **E.** *APOE3*- and *APOE4*-expressing astrocytes were subjected to real-time PCR analysis of *TOMM40* (fold of *APOE3*-expressing cells; n > 3). **F.** Cells were treated with 1 μM MG-132 (MG) for 8 h and the levels of Tom40 were determined by Immunoblot. *Upper panel*, representative results*; Lower panel*, densitometric analysis (fold of *APOE3*-expressing cells; n > 3). **(A-F)** means SE; * *p* < 0.05 and ** *p* < 0.01, *APOE3* compared with *APOE4* cells; ^ *p* < 0.05, treated compared with untreated cells.


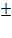


**Figure S3: Levels of mitochondrial proteins and mtDNA in *APOE3/APOE4* astrocytes. A.** *APOE3* and *APOE4* astrocytes were subjected to real-time PCR of *PINK1* expression (fold of *APOE3*-expressing cells; n > 3). **B.** *APOE3* and *APOE4* astrocytes were lysed and subjected to Immunoblot of the indicated mitochondrial proteins *Left panel,* representative results*; right panel*, densitometric analysis (fold of *APOE3*-expressing cells; n > 3) **C.** *APOE3*- and *APOE4* astrocytes were subjected to real-time PCR analysis of the mitochondrial genes *mt-ND1* and *mt-ATP6* (fold of *APOE3*-expressing cells; n > 3). **D**. Cells were treated with 150 nM rapamycin for 72 h, followed by staining with MitoTracker Deep Red (MTDR). Fluorescence intensity of MTDR was measured using flow cytometry (fold of *APOE3*-expressing cells; n > 3). **(A-D)** means SE; ** *p* < 0.01, NS, non-significant,  *APOE3* compared with *APOE4* cells; ^^ *p* < 0.01, treated compared with untreated cells.


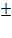

Supplement: Supplementary file 4 — Supplementary Materials and Methods [file 41419_2020_2776_MOESM4_ESM.docx]
